# Shared Neural Correlates Underlying Addictive Disorders and Negative Urgency

**DOI:** 10.3390/brainsci9020036

**Published:** 2019-02-08

**Authors:** Miji Um, Zachary T. Whitt, Rebecca Revilla, Taylor Hunton, Melissa A. Cyders

**Affiliations:** Department of Psychology, Indiana University–Purdue University Indianapolis, Indianapolis, IN 46202, USA; zacwhitt@iu.edu (Z.T.W.); revillarebecca@gmail.com (R.R.); thunton@iu.edu (T.H.); mcyders@iupui.edu (M.A.C.)

**Keywords:** negative urgency, addictive disorders, substance use disorders, pathological gambling, disordered eating

## Abstract

Negative urgency is a personality trait reflecting the tendency to act rashly in response to extreme negative emotions and is considered a transdiagnostic endophenotype for problematic levels of addictive behaviors. Recent research has begun to identify the neural correlates of negative urgency, many of which appear to overlap with neural circuitry underlying addictive disorders associated with negative urgency. The goal of this qualitative review is to summarize the extant literature concerning the neural correlates of negative urgency, to compare these correlates with those implicated with addictive disorders, and to propose new ways to begin to leverage such findings in treatment and intervention approaches. We also address current limitations in the field and make recommendations for areas for future growth in this research domain. Patterns of structure and function in the ventral striatum, frontal regions, such as the prefrontal cortex (PFC) and orbitofrontal cortex (OFC), and amygdala are common across addictive disorders and are related to both real-world risky behaviors and self-report measures of negative urgency. We propose that the time has come to move past considering this trait and these disorders as completely separate entities, and instead for the field to consider how general patterns of convergence across these disorders can lead to a more transdiagnostic approach to treatment and intervention. We suggest future work utilize these convergent patterns in the development of animal models of negative urgency, in the identification and testing of prime pharmacological and physiological interventions, and as objective biomarkers to be used when testing behavioral, pharmacological, and physiological intervention effectiveness. Little empirical work has been done to date in these areas and advances in these nascent fields would advance understanding and applications of the neuroscience of negative urgency.

## 1. Shared Neural Correlates Underlying Addictive Disorders and Negative Urgency

Negative urgency, a personality trait defined as the tendency to act rashly in response to extreme negative emotions [1,2], is one of the personality traits from the UPPS-P model of impulsive behavior, a multidimensional model of impulsivity [3]. The multidimensional model of impulsivity consists of five traits: negative urgency, lack of premeditation (a tendency to act without thinking), lack of perseveration (an inability to stay focused on a task that may be boring or difficult), sensation seeking (a tendency to seek out novel and exciting experiences), and positive urgency (a tendency to act rashly in response to extreme positive emotion). Among them, negative urgency is the personality trait that has been most extensively studied and linked to various addictive disorders, including tobacco use [4,5,6,7], problematic alcohol and drug use [8,9,10], pathological gambling [11], and disordered eating [12]. Therefore, negative urgency is proposed as a transdiagnostic endophenotype for problematic levels of risk-taking behaviors, such as addictive disorders [13].

Because of the clinical implications, recent research has begun to identify the neural correlates of negative urgency in the hopes of designing better treatment and intervention approaches. Existing neuroimaging studies have primarily focused on neural correlates of negative urgency implicated in healthy and various at-risk populations, such as individuals across the drug use spectrum, individuals who engage in pathological gambling, patients with schizophrenia, individuals engaged in risky sexual practices, and individuals with obesity. Most of the previous work has focused on neural correlates of negative urgency using segregated, localized brain regions and has failed to consider functional connectivity or interactions between separate brain regions or large-scale brain networks, which is necessary for more effective pharmacological or physiological treatment design. So far, studies examining negative urgency as related to functional connectivity in the brain are still growing and studies examining how negative urgency relates to structural characteristics or task-based localized blood-oxygen level dependent (BOLD) response are more abundant. Many of the regions identified as related to negative urgency appear to overlap with neurocircuitry underlying addictive disorders. The goal of this qualitative review is to summarize the extant literature concerning the neural correlates of negative urgency and to compare these findings with regions and circuits implicated in addictive disorders. We propose four key ways in which convergent brain evidence for negative urgency and addictive disorders can improve the treatment and intervention process. These proposed areas of future growth are, to date, understudied and require empirical study and support; we hope this review catalyzing research in these domains. 

## 2. Neural Correlates of Negative Urgency

Despite the need to understand how negative urgency relates to addictive disorders in the brain, the neural correlates of negative urgency, in general, are not yet well understood. Here, we review brain regions related to negative urgency from existing neuroimaging work that has investigated negative urgency in the human brain using various imaging modalities. We report those regions that showed converging evidence from at least two or more studies. 

### 2.1. Insula

The insula is involved in emotional decision making [14] and drug addiction (see a review [15]). Also, the insula is a key brain structure for salience network that detects a personally relevant and salient stimulus from a host of stimuli in one’s environment to subsequently act upon [16,17]. One main function of the salience network is visceral interoception [18]. This function is critical to negative urgency, as it may suggest that urgent individuals exhibit aberrant patterns of connections to their own feelings and this may help us understand their rash responses to affective stimuli. The right insula is the most reported region in relation to negative urgency, although some studies also report the bilateral insula as related to negative urgency. In studies with response inhibition tasks, the relationship between negative urgency and insula activation differed as a function of the presence of emotional stimuli, such that the relationship was negative during reward-related response inhibition [19], but positive during negative affect-related response inhibition [20]. Further, negative urgency is related to greater right insula activation during risky decision making in individuals engaging in risky sexual practices [21,22], adolescents who binge drink [23], and healthy young adults [24]. Negative urgency was also related to greater right insula activation in a decision making task when an individual made a risky decision as compared to when the individual previously made a safe decision [24]. Taken together, these findings suggest the context-dependent nature of insula activation in relation to negative urgency. Structurally, negative urgency was related to smaller bilateral insula grey matter volume in a normal weight, but not in an obese group [25]. 

### 2.2. Striatum

The striatum is largely distinguished into two parts: the ventral striatum is activated in response to or in anticipation of reward-related cues, and the dorsal striatum is involved in habitual and compulsive reward seeking [26,27], which signifies the progression of addictive behaviors, especially substance use disorders. Negative urgency seems to be related to both ventral and dorsal parts of the striatum. Greater negative urgency was related to smaller left ventral striatum in healthy participants [28] and lower GABA concentrations in nucleus accumbens (NAcc) in pathological gamblers, which might suggest a relationship between negative urgency and GABA deficiency related to depression and anxiety [29]. Also, negative urgency was related to decreased dopamine receptor binding in the bilateral putamen and caudate [30]. 

Negative urgency moderated the relationship between NAcc feedback signals and response inhibition, such that individuals with low or medium negative urgency showed greater NAcc activation as a function of faster reaction time, but not in those with high negative urgency [19]. In another study, a group of individuals with high levels of negative urgency showed greater activation in the dorsal striatum during response inhibition in the negative affect condition [20]. Similarly, negative urgency was related to greater activation in the left caudate in response to alcohol images among individuals who drink socially [31]. Similar to the findings with the insula, striatum function seems to be context-dependent, as negative urgency is differentially related to regional striatum activation by the presence of emotional or personally relevant stimuli. 

### 2.3. Prefrontal Cortex (PFC)

The prefrontal cortex is related to cognitive control and goal-directed behaviors [32]. Only those high in negative urgency showed greater activation in the dorsolateral PFC (dlPFC) and ventrolateral PFC (vlPFC) in response inhibition during a negative affect condition [20]. Further, negative urgency was related to greater right dlPFC activation during conscious maintenance of negative emotions in a whole sample of cocaine users and controls [33] and greater dlPFC activation during an easy cognitive control task among individuals engaged in risky sexual practices [21]. These findings suggest that lateral prefrontal regions are more intensely recruited during cognitive control when individuals are higher in negative urgency, suggesting these individuals utilize more cognitive resources for tasks involving cognitive control. Also, negative urgency was related to lower GABA concentration in the dlPFC [34]. Negative urgency was related to smaller gray matter volumes in the right vlPFC gray matter volume among individuals with pathological gambling [35] and in the dorsomedial PFC (dmPFC) in healthy controls [28]. 

The orbitofrontal cortex (OFC) contributes to emotion-based learning and decision making [36,37] and is proposed to be a key region in negative urgency [1]. The sub-regions in OFC have distinct functions: the medial OFC (mOFC) is related to evaluation of rewarding stimuli and lateral OFC is related to evaluation of punishing stimuli [38]. Among adolescents who binge drink, greater negative urgency was positively correlated with decreased right OFC activity during an Iowa Gambling task [23]. Negative urgency was related to reduced mOFC/ventromedial PFC (vmPFC) gray matter volume in patients with schizophrenia [39], increased bilateral mOFC/vmPFC activation in response to alcohol odors among social drinkers [40], and weaker mOFC/vmPFC activation in response to high arousal visual stimuli among low sensation seekers [41]. Also, negative urgency was related to greater right lateral OFC activation in response to negative mood images among individuals who drink socially [42] and in response to food odors after satiation among women with obesity, but not in women of normal weight [43]. In sum, negative urgency appears to be related to the activation of the OFC in response to the familiarity and emotional context of stimuli. 

### 2.4. Anterior Cingulate Cortex (ACC) 

The ACC is involved in emotion processing (e.g., emotional saliency) and cognitive processing (e.g., conflict monitoring) [44]. Negative urgency was related to weaker left rostral ACC activations in response to high arousal visual stimuli among individuals low in sensation seeking [41]. In those high in negative urgency, greater dorsal ACC activation was related to better response inhibition performance during a negative affect condition [20]. Also, negative urgency was related to greater left dorsal ACC activation during response initiation (e.g., “Go” trials in a Go/No-Go task) [45].

### 2.5. Amygdala

The amygdala is related to negative emotion processing [46]. The amygdala is thought to be a key region in negative urgency [1] and plays a role in the impulsive system [47]. Negative urgency was related to greater amygdala activation in response to negative mood images [42], and during the reappraisal of negative emotion in individuals who use cocaine and have personality disorders, but not among controls or individuals who use cocaine but are without personality comorbidity [33]. Further, negative urgency was related to lower GABA concentrations in the amygdala among individuals with pathological gambling [29]. 

### 2.6. Temporal Pole

Although its function is less clear, the temporal pole is located between the amygdala and the OFC and anatomically interconnects these regions, which are linked to emotional processing [48]. Olson et al. (2007) theorized the function of the right temporal pole is to link perceptual inputs with visceral emotional responses, which suggests that the region may be related to negative urgency. Negative urgency was related to lower regional gray matter volume in the right temporal pole in healthy controls [28] and lower right middle temporal pole grey matter density across individuals with comorbid cocaine dependence and personality disorders, individuals with cocaine dependence only, and non-drug using controls [49]. 

### 2.7. Frontal Pole

The frontal pole is theorized to synthesize different information to generate goals and goal-related processes and link these processes to specific outcomes to improve future choices [50]. Negative urgency was related to the lower cortical thickness of the right frontal pole in patients with schizophrenia [39] and greater frontal pole activation during an easy cognitive control task among individuals engaged in risky sexual practices [21]. 

### 2.8. Circuit or Network-Level Correlates of Negative Urgency 

While the majority of studies examined negative urgency using structural characteristics of the brain or task-related BOLD responses by separate brain regions, a fewer number of studies started to examine negative urgency-related functional connectivity (FC) or large brain networks. In individuals who use cocaine, negative urgency was more strongly related to FC in the right dlPFC–right insula/OFC during negative emotion maintenance [33]. However, negative urgency was negatively related to the FC in the right inferior frontal gyrus–amygdala during re-appraisal of negative emotions in controls and unrelated in individuals who use cocaine. In another study with individuals who use cocaine, greater negative urgency was related to greater resting-state FC (rsFC) in OFC–subgenual ACC and related to weaker rsFC in right caudate–occipital cortex [51]. Among individuals with alcohol use disorder, greater negative urgency was related to weaker rsFC in the amygdala-striatum network (i.e., impulsive network) [52]. Further, negative urgency was related to weaker rsFC couplings between reflective systems (i.e., OFC network and left executive control network), as well as between the reflective system and the default mode network. In patients with schizophrenia, negative urgency was related to reduced resting-state functional connectivity in left lateral OFC–left middle frontal gyrus, left mOFC–left rostral ACC, and left rostral ACC–left superior/medial frontal gyrus [39]. In adolescents, negative urgency was related to poorer sleep quality, and this relationship was moderated by the FC strength between the default mode network–PFC regions (left vlPFC, left dlPFC) during baseline compared to response inhibition, such that the relationship existed at lower FC strength, but not at the average or high FC strength [53]. In sum, these findings extend the findings from structural and task-based studies by showing that these regions found in such studies do not work individually, but interact or communicate with each other and that negative urgency is also implicated in these interactions.

Recently, Um, Hummer & Cyders [54] conducted seed-based rsFC analyses using seed regions-related to negative urgency (bilateral insula, NAcc, and amygdala) frequently reported in previous studies using tobacco use status. In the whole sample, negative urgency was correlated with greater rsFC strength between the left insula and right dorsal ACC. However, negative urgency was differentially associated with rsFCs in left NAcc–right dACC and in left NAcc–right dlPFC by tobacco use status, such that the relationship was negative in individuals who use tobacco daily and positive in individuals with no lifetime history of tobacco use. This suggests that negative urgency is implicated differentially in negative urgency-related rsFCs, which can provide a preliminary target of intervention for negative urgency-related addictive disorders. 

### 2.9. In Relation to Real-World Behaviors

Some studies showed that negative urgency-related brain regions extend to various real-world behaviors, mainly using mediation models. In one finding, negative urgency mediated the relationship between increased bilateral mOFC/vmPFC activation in response to alcohol odors and subjective craving, as well as problematic alcohol use [40]. Additionally, negative urgency mediated the relationship between greater right lateral OFC and left amygdala activation in response to negative mood images and self-reported risk-taking [42]. In a second finding, among college students, greater right anterior insula activation during response inhibition mediated the relationship between negative urgency and 12-month post-scan alcohol consumption [20]. Among individuals engaged in risky sexual practices, greater right insula, dlPFC, and left frontal pole activation in an easy cognitive control task mediated the relationship between negative urgency and the likelihood of engaging in risky sexual practices in the past 90 days [21]. In sum, these findings suggest that negative urgency-related neural correlates are indeed related to the real-world behaviors that are clinically relevant, supporting the viability of using these correlates as a biomarker for treatment development and effectiveness.

### 2.10. Notes on Positive Urgency

Studies have been heavily focused on negative urgency and neural studies examining positive urgency are less frequent. Of the studies that do exist, the findings are mixed, with a majority of studies reporting null results for positive urgency [20,25,40,42,53,55,56] compared to only three studies reporting null results for negative urgency [57,58,59].

Among the studies that do report neural correlates of positive urgency, findings generally overlap with those of negative urgency. Among patients with schizophrenia, positive urgency was related to lower left rostral ACC and right frontal pole cortical thickness [39]. Among individuals engaged in risky sexual practices, positive urgency, along with negative urgency, was related to increased activity in the right insula during risky sexual decision making [22]. In the easy cognitive control task, individuals who engaged in risky sexual practices showed increased dorsal ACC, dmPFC, and dlPFC activation, and this mediated the positive relationship between positive urgency and the sexual risk-taking [21]. Among adolescents with increased risk for substance use, positive urgency was related to decreased bilateral putamen and inferior frontal gyrus BOLD activation in a rewarded antisaccade task [59]. Positive urgency was related to greater left frontal asymmetry originating from right ACC and medial frontal gyrus [60], as well as from right inferior frontal gyrus [61] in EEG studies. Also, a PET study found that positive urgency was related to decreased dopamine receptor availability in the bilateral NAcc, putamen, and caudate [30]. One study found that urgency (i.e., higher order construct of negative and positive urgency) was related to the greater intensity of low-frequency oscillation in rostral ACC, dorsal ACC, medial frontal gyrus, and right dlPFC [62]. This study also found that greater urgency was related to greater rsFC in poster cingulate cortex/precuneus–midbrain/dorsal striatum. Taken together, the evidence suggests that neural representation of negative and positive urgency likely overlaps, although future studies should examine the extent of overlap between the two urgency traits and whether there is any difference in the strength of the pattern in the overlapping brain regions. 

### 2.11. Converging Evidence for Neural Correlates of Negative Urgency

In summary, recent evidence demonstrates the relationship between negative urgency and brain regions involved in emotion processing (i.e., insula, amygdala, temporal pole, and medial PFCs), specifically that of negative affect, and self-control (i.e., OFC/vmPFC, ACC, frontal pole, and lateral PFCs). Further, reward processing is related to negative urgency (i.e., VS/NAcc, dorsal striatum), which is integral for addictive behaviors. This work provides converging evidence that neural representations for the core components of negative urgency, a tendency to act rashly (i.e., lack of or diminished self-control) under extreme negative emotion, are indeed implicated in the human brain.

Despite the promising converging evidence, there are some notable gaps in this literature. Most existing neuroimaging work has focused on identifying and localizing specific brain regions involved in negative urgency, which does not consider the important effect of interactions between different brain regions. Additionally, the handful of imaging studies that have studied circuit- or network-level neural correlates of negative urgency examined brain regions focused on self-control, a related but broader construct, or brain regions that initially differ between two groups without considering negative urgency levels (with the exception of Um et al., under review [54]). Third, many of these studies have been in small, homogenous clinical samples and have not controlled for the effects of other traits or demographics that might influence these results. Fourth, many of the task-based fMRI studies have dismissed the emotional component of negative urgency, and have the participant undergo response inhibition or decision making-related tasks under neutral emotional states. Providing the context in which participants experience negative emotions is very important for negative urgency to be behaviorally expressed. Thus, existing findings can bias the neural representation of negative urgency, as it may more heavily focus on the cognitive control aspect of the negative urgency without emotional context. Therefore, future studies should examine these neural correlates using a task paradigm that also induces negative emotion. Finally, there has not been any notable cumulative work addressing how these regions might overlap with neurocircuitry identified in a wide variety of addictive disorders. If negative urgency is a transdiagnostic risk factor, we would expect to see the convergence between the neural correlates implicated for negative urgency and those for addictive disorders. 

## 3. Convergence between Brain Correlates of Negative Urgency and Related Addictive Disorders

Here, we briefly summarize the extent neuroscience literature for representative disorders that are related to negative urgency. We focus on disorders that (1) show robust relationships with negative urgency across multiple studies, (2) have a well-established neuroscience literature, and (3) are considered “addictive” in nature. 

### 3.1. Substance Use Disorders

The prominent model of substance use disorders suggests that neurocircuitry of addiction is associated with the three-stage, recurring cycle: from binge/intoxication to withdrawal/negative affect to preoccupation/anticipation/craving [63,64]. These stages involve neuroadaptations in circuits related to various cognitive and emotional processes. The use of drugs increases dopamine neuronal activity in the ventral tegmental area of the mesolimbic dopamine system, which projects to the ventral striatum (VS)/nucleus accumbens (NAcc) [26,27]. Increased dopamine release in the ventral striatum is positively associated with the hedonic effects of drugs and attributes salience to the drugs’ effects, promoting conditioned learning and motivation. The dorsal striatum (caudate and putamen) appears to facilitate the progression from voluntary to compulsive, habitual drug use. Together with prefrontal regions (orbitofrontal, medial, and cingulate) and the amygdala, the dorsal striatum activates in response to drug-related cues and craving. Further, prefrontal regions (dorsolateral and ventrolateral) are important for cognitive control and delayed gratification, which shows dysfunction in drug addiction [65,32]). The hippocampus is related to declarative memory, which involves the retrieval of memories related to contextual cues linked to drug use experiences [66]. The amygdala is also involved in drug addiction through negative reinforcement in drug withdrawal [67]. Anterior regions of the insula are involved in the interoceptive process and drug relapse [68,14]. 

The regions involved in the neurocircuitry of drug addiction highly parallel the regions involved in negative urgency, suggesting that negative urgency is likely related to brain changes across the various stages of the addiction cycle. A larger number of non-neuroimaging studies have already shown that negative urgency is related to various stages in the addiction cycle (e.g., [9] for a review with alcohol use). Negative urgency is involved in the initial phase of drug use as suggested by longitudinal studies that show negative urgency predicts drug use a year later among elementary school students [69,70]. In the brain, negative urgency may be involved in reward processing in the VS/NAcc in the early stages of drug use. Negative urgency is related to greater dependence and problems associated with drug use [9]. In the brain, negative urgency may be involved in the progression to habitual drug use via the dorsal striatum that facilitates this progression, along with other regions related to cue reactivity and emotion processing, such as the OFC, medial PFC, ACC, and amygdala. Negative urgency is associated with various psychopathologies involving cognitive control deficits, including drug addiction [8]. The hyperactivation of the dlFPC and the vlPFC during cognitive processing and reduced structural properties as a function of negative urgency may be related to the cognitive control deficits observed in drug addiction. Further, a recent meta-analysis showed that negative urgency at the start of drug addiction treatment is related to post-treatment relapse [71]. The robust findings of the insula in relation to negative urgency also suggest a role of negative urgency in drug relapse.

### 3.2. Gambling Disorder

Overall, alterations in the prefrontal and reward regions appear to correspond with pathological gambling. Although some recent data show no significant differences in grey matter between individuals with pathological gambling and healthy controls [72], a number of other studies have detected distinct structural differences, including increased grey matter volume in the right middle frontal gyrus, right subcallosal gyrus, left inferior frontal gyrus, and left middle frontal gyrus compared with healthy controls [73]. Conversely, grey matter reductions have been observed in the left supramarginal gyrus, the dorsal part of the left medial orbitofrontal cortex, and the ventral part of the medial orbitofrontal cortex bilaterally (corresponding with loss aversion; [74]), as well as in the superior medial and orbitofrontal cortex [75]. Significant reductions in cortical thickness in the right frontal cortex [76] and bilateral structural grey matter reductions in the posterior cerebellum correspond with pathological gambling [74].

In fMRI studies, individuals with pathological gambling show increased activity in the right lentiform nucleus (including both putamen and globus pallidus) and left middle occipital gyrus relative to healthy controls [77]. Decreased activity in the ventral striatum has been observed for individuals with pathological gambling during tasks related to contemplating potential gain [78]; conversely, increased dopaminergic activity in the ventral striatum correspond with levels of excitement while gambling among those with pathological gambling [79], and dopamine release in the ventral striatum and caudate nucleus has been shown to correspond with pathological gambling symptom severity [80]. Individuals with pathological gambling also demonstrate decreased activity in the ventromedial PFC during anticipation of reward/loss [78], in the insula during anticipation of reward/loss [78], and in the cerebellum [77]. 

Interestingly, pathological gambling is associated with high levels negative urgency [81] and is associated with poor performance on neurocognitive impulse assessments, such as the Iowa Gambling Task [82,83]. The behavior is characterized by an aberrant pattern of cognition related to loss aversion, which previous studies have associated with the functional process of the ventral striatum and amygdala [84,85]. Pathological gambling is also related to dysfunction in the ventral PFC similar to what is seen in substance use disorders [82,86]. It has been proposed that this behavior may be maintained through conditioning related to recall of emotional cues imbedded in the decision-making process of reward/risk-taking behavior [87]. The tendency of individuals with pathological gambling to continue the behavior following significant loss may be related to negative urgency. In comparison to healthy controls, individuals with pathological gambling had higher amounts of dopamine release in the ventral striatum following significant loss [79,88]. 

### 3.3. Disordered Eating

Structurally, differences in grey matter volume have been found in the caudate, dorsal striatum, medial orbitofrontal cortex, hypothalamus, and right lentiform nucleus in individuals with disordered eating [89,90,91]. Neural activation in response to tasting and viewing food occurs differently in individuals with disordered eating patterns as compared to healthy controls [92,93,94] and neural activation is altered in the ventral striatum, insula, medial frontal gyrus, orbitofrontal cortex, anterior cingulate, amygdala, and ventral putamen [95,96,97]. 

Alterations in the neural circuitry of disordered eating are similar to alterations identified in negative urgency. Particularly, disordered eating and negative urgency show similar disruption of several brain areas involved in reward and decision-making processes. These areas include the ventral and dorsal striatum, insula, orbitofrontal cortex, anterior cingulate, and amygdala. The similarities between these brain regions support previous studies that have found a significant association between disordered eating patterns and measurements of negative urgency [98,99,100]. In a study comparing emotion regulation and impulsivity among subtypes of eating disorders, all subtypes reported greater difficulty in emotion regulation as compared with healthy controls [101]. Subtypes included patients with anorexia-restricting type, anorexia-binge purge type, bulimia nervosa, and binge-eating disorder. 

### 3.4. Summary of Convergence and Divergence in Brain Patterns

Overall, we see many regions and circuits that are common across negative urgency and addictive disorders. The first important region is the insula. Activation of the insula has been related to negative urgency [24], and this region shows distinct patterns among individuals with pathological gambling [78], individuals with eating disorders [95,96,97], and has been related to aspects of substance use disorder [68,14]. Second, the striatum is also an area of significant overlap—dopamine release in the striatum creates the hedonic effect experienced during substance use [26,27], and there are important findings concerning the striatum and eating disorders [92,93,94] and pathological gambling symptom severity [80,79]. These findings correspond with structural differences in ventral striatum [28] and differences in neurotransmitter activity in dorsal striatum [30,29] associated with negative urgency. 

Third, the OFC contributes to emotion-based learning and decision making [36,37] and is therefore an important region for negative urgency [23] and associated disorders [40,77]. Structural differences in grey matter concentrations have been observed in the OFC among individuals with pathological gambling [75] and eating disorders [89,90,91]. More generally, the PFC is associated with negative urgency [20,35] and highly involved in the risky-decision making and cognitive failures evident across addictive disorders. Finally, structural differences in the amygdala have been observed across addictive disorders and has been shown to correspond to trait impulsiveness, independent of psychopathology [72]. The amygdala has been shown to be highly implicated in risky-decision making and reward-seeking behaviors [102], making it a key region of interest for negative urgency. 

## 4. Applications to Treatment and Intervention and Suggestions for Future Directions

We suggest that knowledge about these convergent patterns can be very useful for the field. Past research has often focused on the identification of these specific patterns in one particular trait or disorder; we propose that the time has come to move past considering this trait and these disorders as completely separate entities, and instead for the field to consider how general patterns of convergence across these disorders can lead to a more transdiagnostic approach to treatment and intervention. This is important for two reasons. First, most of these disorders are comorbid; therefore, trying to examine pure groups of people with one disorder, but no other, leads to groups that are not likely to represent the real-world experience of a person with these disorders. There is a critical need to understand the complexities of the brain within the context of individuals with complex symptoms and disorders. Second, treatments are often focused on treating one disorder, without consideration of how the presence of other disorders might affect or impede treatment outcomes. Treatment approaches that address comorbid disorder presentations are more likely to be effective in these individuals with complex difficulties.

We propose four key ways in which convergent brain evidence for negative urgency and addictive disorders can improve the treatment and intervention process. Although these proposed ideas are in their infancy, we include them here to catalyze future research to evaluate the viability and feasibility of each of these potential applications. First, our comprehensive review of neuroimaging studies examining negative urgency provides initial evidence that the co-occurrence of negative urgency and these disorders is rooted in the brain, and is not just an artifact of self-report biases. We view negative urgency as a transdiagnostic endophenotype and distal factor not only for addictive behaviors, such as substance use disorders, pathological gambling, and disordered eating, but also various clinical problems that are comorbid with addictive behaviors such as depression, anxiety, personality disorders, schizophrenia, and bipolar disorders [8,103,104,105,106,107,108]. As such, there is a great need for negative urgency to become a critical target in the treatment and intervention process instead of focusing on single or couple disorders [109]. Given its clinical importance and a high relapse rate after drug addiction treatment [110], understanding the neural underpinnings of negative urgency provides a novel avenue for effective treatment development, and we propose that future research be done to design and test such approaches. 

Second, these studies provide prime testable neurobiological underpinnings that can be leveraged as objective biomarkers for the development of novel interventions that directly modify these circuits via pharmacological interventions or transcranial magnetic stimulation approaches, which could be applied to complex comorbid disorder presentations or psychosocial treatment-resistant patients. Specifically, transcranial magnetic stimulation has been examined across various psychopathologies, including depression and schizophrenia, and has shown its effectiveness as a therapeutic tool [111]. The current transcranial magnetic stimulation approaches target a single cortical region to intervene. Many of the key brain regions in negative urgency are subcortical (i.e., amygdala, striatum) or inferior (e.g., temporal pole), which are located in areas that are difficult to directly target using transcranial magnetic stimulation. However, stimulating a single cortical region could stimulate associated brain regions that are hard to directly target [112,113], thereby providing the potential to indirectly target negative urgency-related brain regions. Therefore, cortical regions related to negative urgency, such as the insula, mOFC/vmPFC, ACC, dlPFC, and vlPFC, can work as a target of transcranial magnetic stimulation to co-activate other negative urgency-related regions; this is viable because studies suggest the relationship between negative urgency and the strength of functional connectivity between cortical regions and other key negative urgency-related regions [33,52,53,54]. Neural models can also suggest specific interventions that would not be implicated in traditional behavioral or self-report work. For instance, if research shows that negative urgency is related to amygdala hyper-reactivity (as in [42]), specific interventions that have been shown to buffer amygdala responses to distressing situations (e.g., mindfulness) might be helpful for negative urgency. Further, these biologically-informed interventions can target specific aspects of negative urgency (e.g., bottom-up reactivity), which may prove more or less effective for different urgency phenotypes (e.g., alcohol-dependent individuals) than other approaches (e.g., increasing prefrontally-mediated top-down control). It is our hope that this paper can encourage this sort of scientific inquiry in future work.

Third, in order to test the effectiveness of any newly developed or applied pharmacological or direct stimulation treatment, there is a need to back-translate these findings into an animal model of negative urgency (see a recent review by [114]). There have been a few attempts to do this. A reward omission task was developed to translate and apply negative urgency in a preclinical model of negative urgency using rodents [115,116]. The initial study examined both human and animal models of a reward omission task to represent negative urgency-like behaviors in both species for back-translation [116]. Both humans and rodents learned the association between cues and rewards (monetary rewards for humans, food or drug rewards for rodents) and then learned to respond to the cues (button clicking for humans, lever press for rodents) to receive respective rewards [116]. In both humans and rodents, response rates to the cues increased when the expected rewards were not delivered following the cues. In humans, increased response rates were also related to greater negative urgency scores, suggesting that it might be a behavioral marker for negative urgency that can be back-translated into a preclinical model. A follow-up study with rodents found that the increased response rate following the reward omission was related to greater dopamine transporter (^3^H)DA reuptake in NAcc and serotonin (^3^H)5-HT transporters reuptake in OFC [117], which is consistent with human neuroimaging findings. 

Despite that the reward omission task mirrors negative urgency-like behaviors of humans in rodents and shows potential for preclinical translation of negative urgency, which can then be back-translated to humans, there are a few limitations. First, although the initial study showed the parallel negative urgency-like behaviors between humans and rodents in the reward omission task [116], how the task performance is related to real-world risk-taking behaviors among humans has yet to be examined. Examining the relationship between the task performance and real-world risk-taking behaviors related to negative urgency, such as drug use, would suggest the validity of the task such that the relationship found is not spurious. In turn, it bolsters the argument that the animal model of the same task indeed measures negative urgency-like behaviors and that the findings from the animal model have clinical importance. Second, despite that negative urgency is theorized as a relatively stable personality trait [118,1], the negative urgency-like behavior (i.e., the response rate to expected reward omission) in the rodent model decreased dramatically at later test sessions [117]. The first test session showed the most negative urgency-like behaviors; however, by the third test session, virtually no negative urgency-like behaviors were observed (i.e., the response rate was similar between trials with reward delivery and reward omission). This session effect is concerning because contrary to the idea of negative urgency as a stable trait, the negative urgency-like behaviors in rodents were not stable across multiple test sessions. There might be learning effects, where the rodents learned that there would be the omission of rewards in some trials. and as such, were not as reactive as the first test session. Third, there is a possibility that rodent models of urgency are not feasible, as this phenotype may not exist among animals of immensely different neural articulation. Therefore, future work is needed to examine the validity of the reward omission task producing negative urgency-like behaviors by examining its relationship to real-world risk-taking behaviors with clinical importance and identifying a stable behavioral marker that is aligned to the theoretical conceptualization of negative urgency. Given these limitations in the reward omission task, we suggest that more attempts to develop an animal model of negative urgency should be undertaken, as these models will be prime research tools to identify key compounds and test the effectiveness of such compounds to reduce urgency-related behaviors before applying them to human participants. 

Finally, identification of these neural underpinnings would provide an innovative, objective biomarker to test the effectiveness of negative urgency-based interventions. When negative urgency-related neural correlates are used as objective biomarkers for the development of novel negative urgency-based interventions, these same biomarkers can also work as a method by which to test treatment effectiveness, as they would be the direct targets of intervention pharmacologically, psychosocially, or by stimulation. The objective biomarkers can be tracked without bias in clinical trials, as self-report ratings of clinical symptoms or clinically relevant behaviors can suffer from self-report biases that confound the use of these measures in clinical trials (e.g., hypothesis guessing or placebo reporting effects). Further, this will provide an important initial evidence about the direction of change in these neural correlates as a function of improvement in clinical symptoms and relevant maladaptive behaviors that will guide the advances in negative urgency-based interventions (raising questions such as “Is more or less activation in certain regions related to improvement in negative urgency-related addictive behaviors? Is stronger, weaker, or no connection between brain regions related to improvement in negative urgency-related addictive behaviors?”). This is a prime area of future inquiry.

## 5. Conclusions

It has been well-established that negative urgency is a prime transdiagnostic risk factor for a wide range of addictive disorders [8,13]. Recent neuroimaging data suggest that the neural correlates of negative urgency correspond well with addictive disorders, indicating underlying brain structure and function as important neural markers of these disorders. Common patterns of structure and function in the ventral striatum, frontal regions, such as the PFC and OFC, and amygdala occur across addictive disorders and are related to both real-world risky behaviors and self-report measures of urgency. We propose that the time has come to move past considering negative urgency and these disorders as completely separate entities, and instead for the field to consider how general patterns of convergence across these disorders can lead to a more transdiagnostic approach to treatment and intervention. It is our hope that this qualitative review can catalyze future research into whether or not these convergent patterns can be used in the development of animal models of negative urgency, in the identification and testing of prime pharmacological and physiological interventions, and as objective biomarkers to be used when testing behavioral, pharmacological, and physiological intervention effectiveness. Little empirical work has been done to date in these areas and advances in these nascent fields would advance understanding and applications of the neuroscience of negative urgency.
